# Catquest-9SF questionnaire: validation of Malay and Chinese-language versions using Rasch analysis

**DOI:** 10.1186/s12955-017-0833-3

**Published:** 2018-01-05

**Authors:** Tassha Hilda Adnan, Mokhlisoh Mohamed Apandi, Haireen Kamaruddin, Mohamad Aziz Salowi, Kian Boon Law, Jamaiyah Haniff, Pik Pin Goh

**Affiliations:** 1 National Clinical Research Centre, Kuala Lumpur Hospital, Kuala Lumpur, Malaysia; 2 Ophthalmology Department, Selayang Hospital, Batu Caves, Selangor Malaysia; 3 Clinical Research Centre, Ampang Hospital, Ampang, Selangor Malaysia; 4 Ophthalmology Department, Faculty of Medicine, Sultan Zainal Abidin University, Kuala Terengganu, Terengganu Malaysia

**Keywords:** Validation, Catquest-9SF questionnaire, Rasch analysis

## Abstract

**Background:**

Catquest questionnaire was originally developed in Swedish to measure patients’ self-assessed visual function to evaluate the benefit of cataract surgery. The result of the Rasch analysis leading to the creation of the nine-item short form of Catquest, (Catquest-9SF), and it had been translated and validated in English. The aim is therefore to evaluate the translated Catquest-9SF questionnaire in Malay and Chinese (Mandarin) language version for measuring patient-reported visual function among cataract population in Malaysia.

**Methods:**

The English version of Catquest-9SF questionnaire was translated and back translated into Malay and Chinese languages. The Malay and Chinese translated versions were self-administered by 236 and 202 pre-operative patients drawn from a cataract surgery waiting list, respectively. The translated Catquest-9SF data and its four response options were assessed for fit to the Rasch model.

**Results:**

The Catquest-9SF performed well in the Malay and Chinese translated versions fulfilling all criteria for valid measurement, as demonstrated by Rasch analysis. Both versions of questionnaire had ordered response thresholds, with a good person separation (Malay 2.84; and Chinese 2.59) and patient separation reliability (Malay 0.89; Chinese 0.87). Targeting was 0.30 and −0.11 logits in Malay and Chinese versions respectively, indicating that the item difficulty was well suited to the visual abilities of the patients. All items fit a single overall construct (Malay infit range 0.85–1.26, outfit range 0.73–1.13; Chinese infit range 0.80–1.51, outfit range 0.71–1.36), unidimensional by principal components analysis, and was free of Differential Item Functioning (DIF).

**Conclusions:**

These results support the good overall functioning of the Catquest-9SF in patients with cataract. The translated questionnaire to Malay and Chinese-language versions are reliable and valid in measuring visual disability outcomes in the Malaysian cataract population.

## Background

Cataract is a disease due to lens opacity and the leading cause of blindness in the developing nations, and as such is a major public health issue [1]. It was found to be the leading cause of blindness and second commonest cause of visual impairment after uncorrected refractive error in Malaysia [2]. The main goal of treating cataract is to improve visual acuity and therefore the visual function, considering that it entails improvements in quality of life. The ophthalmologist presumes that the post-operative visual acuity is an objective measure for their patients’ satisfaction. However, improvements in such measures after surgery can produce small effects or may be still associated with little or a decline in quality of life especially if the adverse event of the surgery is high or there is presence of ocular or systemic morbidity. In one end of the spectrum, a study by Naiem et al., cataract surgery is cost-effective even in a subpopulation of patient with a lower (less than 30%) predicted probability of reporting improved visual functioning after surgery at whom a strategy of watchful waiting may be equally effective and considerably less expensive [3].

Determining the success of the surgery is of the utmost importance. In clinical practice, visual acuity and residual refractive error are generally used to quantitatively measure the patient’s visual function from the surgeon’s perspective. However, it is the patient’s self-perceived visual function and the improvement in the quality of life that is gradually recognized by clinical practitioners, i.e. the ability to perform day-to-day tasks is one of the most important outcomes from the patient’s perspective. Indeed, vision-related quality of life is the issue of greatest importance to many cataract patients, as it reduces a person’s ability to perform activities of daily living, increases risk of depression, falls and hip fractures, and impairs their quality of life [4–6]. It is also important to note that some patients, who have had uneventful cataract surgery with improvement in visual acuity, may still be dissatisfied with their quality of life [7]. Assessment of quality of life provides a holistic view of the impact following the cataract surgery on a patient’s life from the patient’s perspective. Additionally, it helps in micro and macro level in aiding health care decision making and optimal performance to improve patient care and outcome.

Patient-reported outcomes (questionnaires) have become an essential component to assess visual functioning of cataract surgery, beyond the results of a clinical evaluation [8]. There are a few questionnaires that have been developed to assess visual disability in cataract patients, and revalidated using Rasch analysis, for example Quality of life and Vision Function Questionnaire (QOL-VFQ), Activities of Daily Vision Scale (ADVS), and Cataract TyPE Specification (TyPE Spec) [9–11].

The Catquest questionnaire was developed to measure patients’ self-assessed visual function to evaluate the benefit of cataract surgery [12]. The items in Catquest were grouped into four dimensions: frequency of performing activities (6 questions), perceived difficulty in performing daily-life activities (7 questions), global questions about difficulties in general and satisfaction with vision (2 questions), and cataract symptoms (2 questions). Each item has four response categories. The original Catquest questionnaire has been assessed using Rasch analysis. The result of these analysis lead to the creation of a nine-item short form of Catquest, the Catquest-9SF, which was shown to be highly responsive to surgical treatment and the score was moderately correlated with visual acuity [13].

The Catquest questionnaire was originally developed in Swedish and was translated and validated in an English-speaking population in Australia [14]. While in Malaysia with a total population of approximately 28.3 million based on a 2010 population report and as a multiracial country, the Malay race makes up the majority of the population (63.1%) followed by the Chinese (24.6%), Indians (7.3%), and others (5.0%). Malays and Chinese not only boast the largest population in Malaysia, but also as the highest population with cataract surgeries, there were 43.2% Malays and 33.7% Chinese, according to the Malaysian Cataract Surgery Registry data from 2002 to 2012 [15]. The aim of this paper is therefore to translate and validate the Malay and Chinese-language versions of the Catquest-9SF questionnaire for cataract population in Malaysia.

## Methods

### The Catquest-9SF questionnaire and the translation

The Catquest-9SF is a Rasch-scaled questionnaire consisting of nine items measuring activity limitation in patients’ daily life because of the vision before and after their cataract surgery. It comprises 2 global assessment questions about the patients’ difficulties in general and their satisfaction with the vision, and 7 questions of perceived difficulty in performing daily-life activities. Each item has four response options: 4 = ‘Yes, very great difficulties’; 3 = ‘Yes, great difficulties’; 2 = ‘Yes, some difficulties’; and 1 = ‘No, no difficulties’. For the global question about the patients’ satisfaction with their vision, the response categories are as follows: 4 = ‘Very dissatisfied’; 3 = ‘Rather dissatisfied’; 2 = ‘Fairly satisfied’; and 1 = ‘Very satisfied’. All of the items contain an additional option of ‘Cannot decide’ which is considered as missing in the Rasch analysis.

The Catquest-9SF questionnaire was translated into two languages: Malay and Chinese (Mandarin), by two bilingual translators consisting of a-blinded linguistic expert from the Malaysian National Institute of Translation (ITNM Berhad) and non-blinded ophthalmologist/optometrist, native to Bahasa Melayu or Chinese (Mandarin) for each type of translation. The two translated versions were compared, and the discrepancies between translators were resolved. The Malay and Chinese versions of the Catquest-9SF were then back-translated to English by two lay translators working independently. The investigators reviewed and compared the back-translation with the English version of the Catquest-9SF to reach consensus of the pre-final versions. The pre-final versions were tested in series of 8 to 10 focus group participants. These participants were lay persons who speak bilingual Malay-English or Mandarin-English who commented on the translated versions and formats for each of the Malay and Mandarin respectively. Later, the investigators reviewed and edited the results of all the discussions to form and produced the final version.

### Participants

Participants were recruited from the cataract surgery waiting list of three hospitals in Malaysia, namely Selayang Hospital, Serdang Hospital and Hospital Umum Sarawak, between October 2012 and March 2013. The Malay and Chinese translations of Catquest-9SF were administered to patients of 18 years and above with no severe cognitive impairment during their pre-clerking visit in the eye clinic or on operative day prior to surgery. Typical of a cataract population, patients with co-existing ocular and systemic co-morbidities were included. The questionnaire was self-administered to Malay or Chinese-speaking and writing people, however, if participants could understand the language but have bilateral dense cataracts which can cause difficulty in reading and writing, a close relative could help to complete the questionnaire. All questionnaires were completed and returned on the same day during the clinic visit, before the cataract surgery. The information regarding the co-morbidities was then verified by the treating ophthalmologist in the medical records.

### Rasch analysis

A Rasch analysis compares the level of difficulty required to perform a task as listed in the items with the person’s level of ability to perform that task, and both are sorted in the same linear scale. The ordinal raw score of the data were transformed into a unit known as the logit in the Rasch model, which is the natural logarithm of the odds ratio.

The analyses of Catquest-9SF data for both Malay and Chinese-language versions were performed with the Winsteps software (version 3.72.3) [16, 17] using the Andrich rating scale model for polytomous data. The psychometric properties of the cataract patient-reported outcome questionnaire were assessed with behaviour of the rating scale (or category threshold), fit statistics, separation indices, and targeting. The first step was an assessment of the behaviour of the rating scale. The rating scale test explores whether the three category thresholds (since Catquest-9SF uses four response categories, omitting ‘Cannot decide’ as missing) between the response probabilities, are ordered.

Second, the ability of the Catquest-9SF of both versions to discriminate different strata (or groups) among participants was assessed using person separation and the separation reliability. The separation reliability coefficient represents the precision of the item measures. A larger reliability values (ranges from 0 to 1) indicate a higher ability to distinguish between the strata of person ability. A minimum person separation of 2.0 and reliability of 0.8 corresponded to the ability to differentiate among three strata [10].

Third, overall fit of the data to the model was assessed using two fit statistics: infit and outfit mean square. When the data fit well, they indicate that the items contributed to a single underlying construct (unidimensionality). Both infit and outfit mean squares have an expected value of 1, with acceptable fit criterion of 0.7 to 1.3.

Fourth, we checked the targeting precision to determine whether the questionnaire items were appropriate to the studied population of people with cataract. Targeting is assessed by the pattern of the distributions appearing on a person–item map and by the difference in the value of the person and item mean scores. For a well-targeted instrument, the ability of the patients and the difficulty of the questions should centre on the same mean (targeting of 0). In general, a difference between the mean person and item score of more than 1.0 logit indicates notable mistargeting [18]. A person–item map also visualized the item hierarchy of difficulty, ranging from least to most difficult to perform.

In addition to item-fit statistics, unidimensionality was assessed further using principal components analysis (PCA) of the residuals. Unidimensionality provides evidence that the instrument measure the underlying trait (visual difficulty). The assessment is performed by the comparison of the amount of variance explained empirically and by the model, and the amount of variance explained by the first contrast (additional dimension).

Finally, we assessed differential item functioning (DIF) to determine the differences between the various item difficulty estimates among patients. DIF allows comparison of each item calibration between groups, stratified by age (≤65 years vs >65 years; 65 years was the mean age of our sample), gender and visual acuity group in order to indicate whether different groups have systematically different responses to Catquest-9SF items. The cut-off of 65 years old was also consistent with the mean age reported for patients with cataract surgery done in Malaysia, as captured in the 6^th^ Report of the National Eye Database 2012 [19]. DIF was considered absent if the magnitude was less than 0.50 logits, larger than 0.50 to 1.00 logits was treated as substantial, and significant DIF as values greater than 1.00 logits.

## Results

### Participant characteristics

A total of 438 patients completed the translated Catquest-9SF questionnaire (Table 1). Overall, the participants’ mean age was 63 years (SD = 10.1) and 68 years (SD = 9.0) for Malay and Chinese version, respectively; and about 54% were female. Using best-corrected data in the better-seeing eye, the majority of the Malay version participants had low vision (44.1%) with 1.7% (4/236) blindness, as well as majority of 45.5% with low vision and 2.0% (4/202) with blindness among participants of Chinese version. For the Malay version, 26.7% patients had pre-existing ocular comorbidity and 76.3% with systemic comorbidity; while for Chinese version, 36.6% and 67.8% patients had pre-existing ocular or systemic comorbidity, respectively. From our cohort, the pre-existing ocular co-morbidities include glaucoma, retinal disease, corneal disease, pterygium, proliferative diabetic retinopathy, non-proliferative diabetic retinopathy and others, excluding cataract since majority all the patients’ presence with cataract conditions. The majority of Malay version questionnaire was completed by the Malay participants (76%), while the Chinese version was all completed by the Chinese participants (100%). Most of the participants in our cohort had finished their primary school to higher education (78.4% of Malay version and 74.3% of Chinese version). Table 2 shows the responses for the items included in the Catquest-9SF questionnaire.

**Table 1:** Characteristics of study population

	Malay version (*n* = 236)		Chinese version (*n* = 202)	
	n	(%)	n	(%)
Age (years):				
Mean (SD)	63 (10.1)		68 (9.0)	
Minimum, maximum	21, 84		29, 86	
Gender:				
Male	107	(45.3)	93	(46.0)
Female	129	(54.7)	109	(54.0)
Race:				
Malay	179	(75.8)	0	(0.0)
Chinese	2	(0.8)	202	(100.0)
Indian	31	(13.1)	0	(0.0)
Others	24	(10.2)	0	(0.0)
Education level:				
No formal education	49	(20.8)	51	(25.2)
Primary	88	(37.3)	101	(50.0)
Secondary	82	(34.7)	44	(21.8)
Higher education	15	(6.4)	5	(2.5)
Best corrected visual acuity:				
6/6–6/15	76	(32.2)	79	(39.1)
6/18–3/60	104	(44.1)	92	(45.5)
2/60 – NPL	4	(1.7)	4	(2.0)
Ocular comorbidity^a^	63	(26.7)	74	(36.6)
Systemic comorbidity^b^	180	(76.3)	137	(67.8)
Answering questionnaire:				
Self-administered	77	(32.6)	62	(30.7)
Assistant administered	157	(66.5)	139	(68.8)

Number and column percentages are based on available information; the remaining unreported percentage is missing value

*SD* Standard deviation, *NPL* No perception of light

^a^Includes cornea disease, glaucoma, retina disease, non-proliferative diabetic retinopathy, proliferative diabetic retinopathy, pterygium, others

^b^Includes diabetes mellitus, hypertension, heart disease, hyperlipidaemia, others

**Table 2:** Response of the items included in the Catquest-9SF questionnaire

Item	Malay version (*n* = 236)		Chinese version (*n* = 202)	
	n	(%)	n	(%)
^a^1. Do you experience that your present vision gives you difficulties in any way in your daily life?				
Yes, very great difficulties	52	(22.0)	19	(9.4)
Yes, great difficulties	75	(31.8)	54	(26.7)
Yes, some difficulties	82	(34.7)	111	(55.0)
No, no difficulties	26	(11.0)	17	(8.4)
Cannot decide	1	(0.4)	1	(0.5)
^a^2. Are you satisfied or dissatisfied with your present vision?				
Very dissatisfied	63	(26.7)	58	(28.9)
Rather dissatisfied	95	(40.3)	95	(47.3)
Fairly satisfied	61	(25.8)	38	(18.9)
Very satisfied	17	(7.2)	7	(3.5)
Cannot decide	0	(0.0)	3	(1.5)
Do you have difficulty with the following activities because of your vision?				
1 Reading text in the newspaper				
Yes, very great difficulties	77	(32.6)	42	(20.8)
Yes, great difficulties	56	(23.7)	56	(27.7)
Yes, some difficulties	68	(28.8)	82	(40.6)
No, no difficulties	21	(8.9)	15	(7.4)
Cannot decide	14	(5.9)	7	(3.5)
2 Recognizing faces of people you meet				
Yes, very great difficulties	33	(14.0)	20	(9.9)
Yes, great difficulties	65	(27.5)	38	(18.8)
Yes, some difficulties	76	(32.2)	83	(41.1)
No, no difficulties	60	(25.4)	60	(29.7)
Cannot decide	2	(0.9)	1	(0.5)
3 Seeing prices of goods when shopping				
Yes, very great difficulties	74	(31.4)	40	(19.8)
Yes, great difficulties	56	(23.7)	59	(29.2)
Yes, some difficulties	55	(23.3)	82	(40.6)
No, no difficulties	44	(18.6)	16	(7.9)
Cannot decide	7	(3.0)	5	(2.5)
4 Seeing to walk on uneven ground				
Yes, very great difficulties	48	(20.4)	17	(8.4)
Yes, great difficulties	64	(27.2)	35	(17.3)
Yes, some difficulties	70	(29.8)	104	(51.5)
No, no difficulties	50	(21.3)	46	(22.8)
Cannot decide	3	(1.3)	0	(0.0)
5 Seeing to do needlework and handicraft				
Yes, very great difficulties	55	(23.3)	28	(14.0)
Yes, great difficulties	53	(22.5)	45	(22.5)
Yes, some difficulties	62	(26.3)	88	(44.0)
No, no difficulties	44	(18.6)	25	(12.5)
Cannot decide	22	(9.3)	14	(7.0)
6 Reading text on television				
Yes, very great difficulties	56	(23.7)	23	(11.4)
Yes, great difficulties	55	(23.3)	69	(34.2)
Yes, some difficulties	66	(28.0)	74	(36.6)
No, no difficulties	44	(18.6)	30	(14.9)
Cannot decide	15	(6.4)	6	(3.0)
7 Seeing to carry out a preferred hobby				
Yes, very great difficulties	36	(15.2)	19	(9.4)
Yes, great difficulties	58	(24.6)	38	(18.8)
Yes, some difficulties	67	(28.4)	99	(49.0)
No, no difficulties	66	(28.0)	37	(18.3)
Cannot decide	9	(3.8)	9	(4.5)

Number and percentages are based on available information

^a^Assessment items

### Rasch analysis

**Fig. 1:**
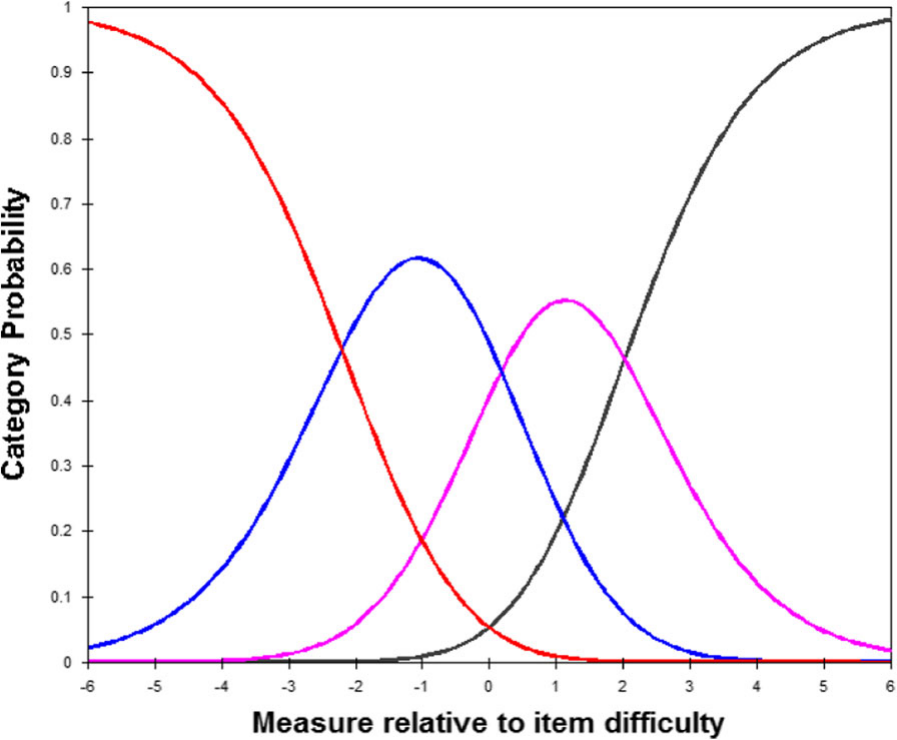
Category Probability curve for the 9-items of Malay-language version

**Fig. 2:**
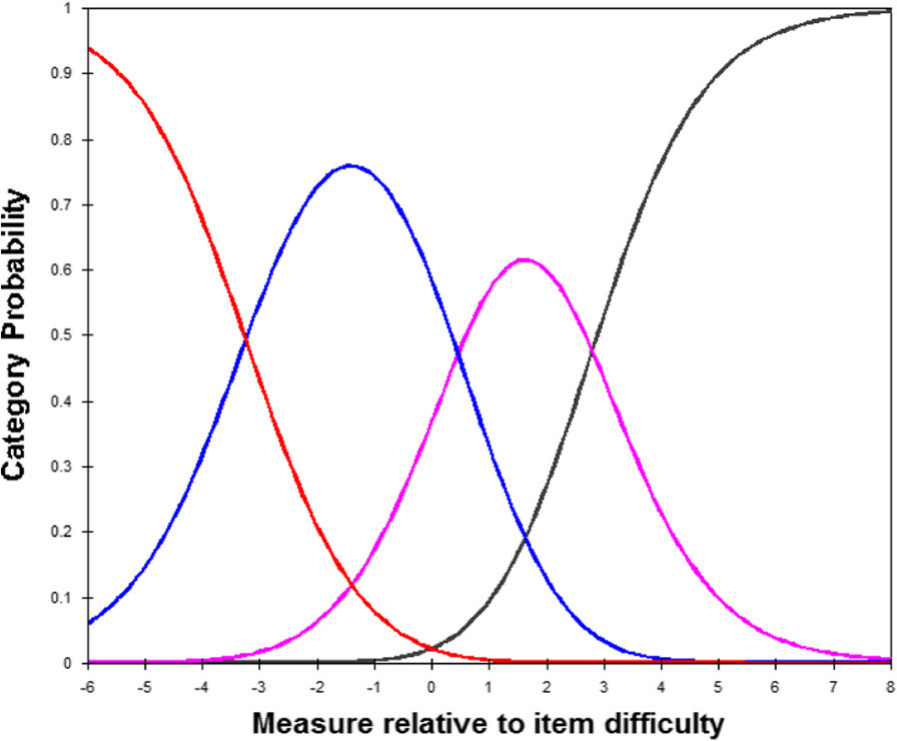
Category probability curve for the 9-items of Chinese-Language version

The category probability curves for the 9-items in Catquest-9SF (Figs. 1 and 2) illustrates that the category thresholds were ordered, indicating the original rating scale functioned well for both versions. The overall performance of both translated versions was acceptable with satisfactory fit to the Rasch model (Table 3). A high measurement reliability and high separation index for the 9-items demonstrated the capacity to distinguish between three strata (or groups) of patient ability. Nevertheless, excluding the 2 global assessment questions of the Catquest, there was still an adequate person separation with a ratio of 2.59 in Malay version and 2.30 in Chinese version (which is greater than the 2.0), and adequate person reliability of 0.87 and 0.84, respectively (which is greater than the 0.80). Table 4 shows that all items fit a single overall construct for the Malay version (infit range 0.85–1.26; outfit range 0.73–1.13), and Chinese version (infit range 0.80–1.51; outfit range 0.71–1.36).

**Table 3:** Summary statistics for person and item parameters of the Catquest-9SF

Version		Parameter	Separation index	Reliability	Average infit mean square	Average outfit mean square
Malay	9-items	Person ability	2.84	0.89	1.00	1.01
		Item difficulty	4.81	0.96	1.00	1.00
	7-items^a^	Person ability	2.59	0.87	1.00	0.99
		Item difficulty	4.97	0.96	1.00	0.98
Chinese	9-items	Person ability	2.59	0.87	1.00	1.01
		Item difficulty	6.96	0.98	1.00	1.02
	7-items^a^	Person ability	2.30	0.84	1.01	0.98
		Item difficulty	5.28	0.97	1.00	0.98

^a^7-items excluding 2 global assessment questions

**Table 4:** Item fit statistics for the Catquest-9SF

Item	Malay version					Chinese version				
	Item calibration^a^ (SE)	Infit		Outfit		Item calibration^a^ (SE)	Infit		Outfit	
		MNSQ	ZSTD	MNSQ	ZSTD		MNSQ	ZSTD	MNSQ	ZSTD
Assessment items:										
1. Do you experience that your present vision gives you difficulties in any way in your daily life?	−0.16 (0.11)	0.97	−0.3	0.99	−0.1	0.19 (0.13)	1.09	0.9	1.10	1.0
2. Are you satisfied or dissatisfied with your present vision?	−0.77 (0.11)	1.26	2.6	1.51	4.1	−2.05 (0.13)	1.01	0.2	1.36	2.3
Do you have difficulty with the following activities because of your vision?										
1. Reading text in the newspaper	−0.81 (0.11)	0.85	−1.6	0.81	−1.8	−0.66 (0.13)	1.12	1.2	1.09	0.9
2. Recognizing faces of people you meet	0.83 (0.11)	1.02	0.3	1.01	0.1	1.18 (0.14)	1.13	1.3	1.12	1.1
3. Seeing prices of goods when shopping	−0.34 (0.11)	1.09	0.9	1.04	0.5	−0.67 (0.13)	0.93	−0.7	0.91	−0.8
4. Seeing to walk on uneven ground	0.34 (0.11)	1.01	0.2	1.01	0.1	1.12 (0.14)	1.06	0.6	0.97	−0.2
5. Seeing to do needlework and handicraft	0.03 (0.12)	1.01	0.2	0.97	−0.2	0.11 (0.14)	0.97	−0.2	0.93	−0.6
6. Reading text on television	0.07 (0.11)	0.89	−1.1	0.85	−1.5	−0.01 (0.13)	0.98	−0.1	0.97	−0.3
7. Seeing to carry out a preferred hobby	0.82 (0.11)	0.86	−1.5	0.80	−2.0	0.79 (0.14)	0.73	−2.8	0.71	−2.9

*SE* standard error, *MNSQ* mean square, *ZSTD* standardized fit statistic

^a^Measured in logit; positive item logit indicates that the item requires a lower visual ability than the mean of the items and is an easier item; while a negative item logit indicates that the item requires a higher visual ability than the mean of the items and is a more difficult item

Unidimensionality by PCA of the residuals for the Malay version showed that the variance explained by the measures was comparable for the empirical calculation (63.5%) and by the model (63.5%). The unexplained variance by the first contrast was 2.0 eigenvalue units (8.0%), which is close to the magnitude seen with random data. The two global assessment items (i.e. “Do you experience that your present vision gives you difficulties in any way in your daily life?” and “Are you satisfied or dissatisfied with your present vision?”) correlated with the first contrast (visual difficulty in general: 0.78 and satisfaction with vision: 0.71). The Cronbach’s α was 0.93. For the Chinese version, the variance explained by the Rasch measure was 60.4% which is also comparable with the expected by the model (60.5%). The unexplained variance in the first contrast was 2.0 eigenvalue units (8.8%). The two global assessment items correlated with the first contrast (visual difficulty in general: 0.64 and satisfaction with vision: 0.47), and the Cronbach’s α was 0.94.

**Fig. 3:**
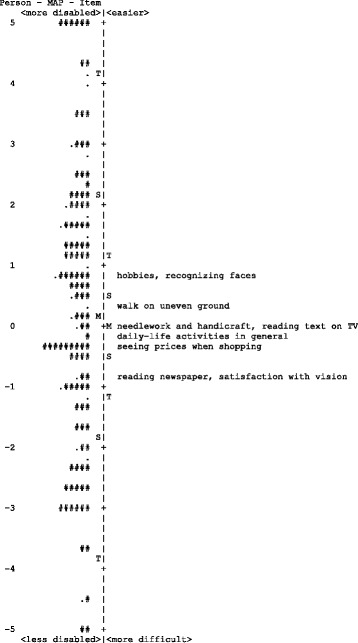
Person-item map of Malay-language version of Catquest-9SF The participants are located on the left of the dashed line, with less disabled participants located at the bottom of the map. Items are located on the right of the dashed line and more difficult items are located at the bottom of the map. Each ‘#’ represents 2 participants and each ‘.’ represents 1 participant. (M = mean; S = 1 standard deviation; T = 2 standard deviations)

**Fig. 4:**
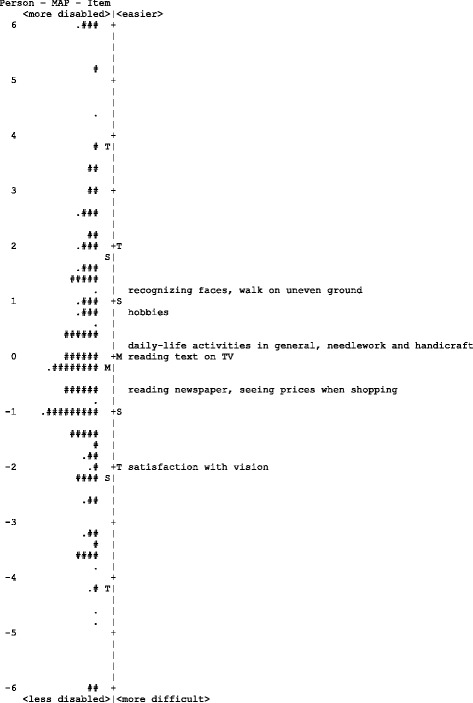
Person-item map of Chinese-language version of Catquest-9SF The participants are located on the left of the dashed line, with less disabled participants located at the bottom of the map. Items are located on the right of the dashed line and more difficult items are located at the bottom of the map. Each ‘#’ represents 2 participants and each ‘.’ represents 1 participant. (M = mean; S = 1 standard deviation; T = 2 standard deviations)

The person-item map given in Figs. 3 and 4 illustrates the relationship between item difficulty and person ability. There was a fairly even spread of items along the variable and the person ability which demonstrated a 11.27 logits spread (−5.72 to 5.55 logits; mean = 0.30) in Malay version and 13.62 logits spread (−7.10 to 6.52 logits; mean = −0.11) in Chinese version. For Malay version, item difficulty demonstrated a 1.64-logit spread (−0.81 to 0.83 logits), the two easiest items were recognizing faces (0.83) and performing a hobby (0.82), and the two most difficult items were reading the newspaper (−0.81) and satisfaction with vision (−0.77). While for Chinese version, item difficulty demonstrated a 3.23-logit spread (−2.05 to 1.18 logits), the two easiest items were recognizing faces (1.18) and seeing to walk on uneven ground (1.12), and the most difficult items were reading the newspaper (−0.66), seeing prices of goods when shopping (−0.67), and satisfaction with vision (−2.05).

Table 5 shows that the Catquest-9SF was free of DIF, indicating that the items functioned similarly across different subgroups of patients (stratified by age group, gender and visual acuity group), for both Malay and Chinese version. Therefore, the Malay and Chinese Catquest-9SF questionnaire showed good person separation, well-targeted and unidimensionality and was free of any large DIF.

**Table 5:** Differential item functioning (DIF) by age, gender and visual acuity

Version	Item	DIF by age				DIF by gender				DIF by VA			
		Item difficulty		Diff in logits	*P*-value^a^	Item difficulty		Diff in logits	*P*-value^a^	Item difficulty		Diff in logits	*P*-value^a^
		≤65 years	>65 years			Male	Female			VA >6/18	VA ≤6/18		
Malay	*n*=	141	94			107	129			76	108		
	Daily-life activities in general	−0.16	−0.20	0.04	0.866	−0.16	−0.16	0.00	>0.995	−0.49	0.01	−0.51	0.043
	Satisfaction with vision	−0.88	−0.66	−0.22	0.341	−0.77	−0.77	0.00	>0.995	−0.71	−0.87	0.16	0.515
	Reading newspaper	−0.77	−0.88	0.11	0.649	−0.98	−0.67	−0.31	0.181	−0.77	−0.84	0.17	0.778
	Recognizing faces	0.86	0.78	0.08	0.724	0.90	0.77	0.13	0.561	1.13	0.55	0.58	0.029
	Seeing prices when shopping	−0.27	−0.43	0.16	0.486	−0.43	−0.27	−0.15	0.498	−0.46	−0.34	−0.12	0.626
	Walk on uneven ground	0.34	0.39	−0.05	0.815	0.53	0.18	0.35	0.118	0.45	0.22	0.23	0.369
	Needlework and handicraft	0.06	0.03	0.02	0.916	0.03	0.03	0.00	>0.995	0.07	0.23	−0.16	0.551
	Reading text on TV	−0.01	0.20	−0.20	0.382	−0.03	0.15	−0.18	0.432	0.11	0.21	−0.10	0.685
	Hobbies	0.85	0.78	0.07	0.772	0.87	0.78	0.08	0.712	0.72	0.82	−0.10	0.717
Chinese	*n*=	70	131			93	109			79	96		
	Daily-life activities in general	−0.01	0.28	−0.29	0.299	0.36	0.04	0.32	0.230	−0.03	0.24	−0.26	0.360
	Satisfaction with vision	−2.19	−1.98	−0.21	0.465	−2.16	−1.95	−0.21	0.443	−2.12	−2.05	−0.07	0.809
	Reading newspaper	−0.73	−0.64	−0.09	0.748	−0.54	−0.77	0.23	0.377	−0.66	−0.61	−0.05	0.856
	Recognizing faces	1.30	1.13	0.17	0.568	0.92	1.40	−0.48	0.086	1.44	1.02	0.42	0.163
	Seeing prices when shopping	−0.71	−0.67	−0.04	0.895	−0.79	−0.56	−0.23	0.384	−0.57	−0.67	0.10	0.724
	Walk on uneven ground	1.12	1.12	0.00	>0.995	1.17	1.09	0.08	0.760	0.98	1.21	−0.23	0.439
	Needlework and handicraft	0.14	0.11	0.03	0.927	0.25	−0.01	0.26	0.345	0.04	0.28	−0.24	0.419
	Reading text on TV	0.21	−0.12	0.32	0.259	−0.01	−0.01	0.00	>0.995	0.17	−0.13	0.30	0.300
	Hobbies	0.89	0.75	0.14	0.635	0.81	0.79	0.02	0.942	0.79	0.71	0.08	0.793

^a^Welch’s test

## Discussion

The Rasch analysis result shows that both translated Malay and Chinese Catquest-9SF versions are reliable, valid and unidimensional measures to assess the visual functioning in Malaysian cataract patients. The translated version met the requirements of the Rasch model and our results also prove that the Catquest-9SF scale has ordered thresholds and is well-targeted, and were free of any large DIF, as confirmed in previous studies of Swedish and Australian cataract patients [13, 14].

The person separation index as a measure of reliability was high, suggesting good discriminant ability of the questionnaire among three distinct strata of participants, i.e. good, intermediate, and poor. The category rating scale of the Catquest-9SF worked well, and the patients could use the scale to differentiate the four levels of items difficulty.

The person-item map enables visualization of patient ability and item difficulty, and there was a fairly even spread of items about the ability continuum. The items were well-targeted to the subjects, with a mean difference of 0.30 and −0.11 logits in Malay and Chinese versions, respectively. This means that the difficulty of the items on the translated questionnaire were appropriate for the ability of patients. However, a slight mistargeting persisted in the Australian and Chinese studies, indicating that the items were found to be easy for the visual abilities of the patients [14, 20]. Similar to the findings in the Swedish, Australian and Chinese cohort in China, item “Satisfaction with the present vision” was found to be the most difficult question for Malay and Chinese versions. “Recognizing faces” was the easiest item in both versions, comparable to Swedish and Australian cataract patients [13, 14, 20].

There was no large DIF for any of the items in the Malay version, indicating that the items were behaving similarly across different subgroups of patients, i.e. by age, gender and visual acuity group. There was also no large DIF reported for the majority of the items in the Chinese-translated version, dissimilar with the findings in China study where the item “Reading text on TV” showed significant DIF across age and gender groups. The China study found that the older people of more than 60 years and women were more likely to experience the difficulty in that item [20]. The Swedish and Australian study found that women rated the item “Seeing to walk on uneven ground” easier than men [13, 14].

The Catquest-9SF questionnaire is one of the instruments recommended by Lundström & Pesudovs (2011) in their study of reviewing instruments for measuring the vision-related activity limitation for cataract surgery outcomes. The justification is due to its psychometric properties, targeting, and being a short tool [21]. It is relatively easy, understandable, also cost and time-effective to administer encouraging a high rate of responses from the participants. However, the limitation of our study is the cohort only comprised preoperative cataract patients, thus the responsiveness of the questionnaires (i.e. the change in visual disability) after the cataract surgery was not tested.

## Conclusions

It is notable that the findings derived from the study showed excellent psychometric characteristics. In conclusion, the study demonstrated that the translation of the Catquest-9SF questionnaire into Malay and Chinese languages were easily understood and practical, and proven to be a reliable and valid instrument for the assessment of visual function of cataract patients. Consequently, it may be useful and can be administered in routine clinical use aiming to assess vision function of patients with cataract in Malay and Chinese-speaking communities in Malaysia.
